# Kinetics of improved 1,4-alpha-D-glucan glucohydrolase biosynthesis from a newly isolated Aspergillus oryzae IIB-6 and parameter significance analysis by 2-factorial design

**DOI:** 10.1186/2193-1801-1-32

**Published:** 2012-10-06

**Authors:** Bilqees Fatima, Sikander Ali

**Affiliations:** Institute of Industrial Biotechnology (IIB), GC University Lahore, H.30, St.7, Tezab Ahata, Lahore-39, Pakistan

**Keywords:** Aspergillus oryzae, Batch-culture, 2-factorial design, Glucoamylase, Kinetics, Mould culture

## Abstract

Sixteen different mould cultures viz. *Aspergillus*, *Alternaria*, *Arthroderma, Trichoderma, Fusarium, Penicillium, Rhizopus* and *Chochliobolus* were isolated from the soil samples of Qatar by serial dilution method. The preliminary screening of isolates was done by selecting initial colonies showing relatively bigger zones of starch hydrolysis on nutrient agar plates. The isolates were then subjected to secondary screening by submerged fermentation (SmF). The 1,4-α-D-glucan glucohydrolase (GGH) activity ranged from 1.906-12.675 U/ml/min. The product yield was analysed in dependence of mycelial morphology, biomass level and protein content. The isolate *Aspergillus oryzae* llB-6 which gave maximum enzyme production was incubated in M3 medium containing 20 g/l starch, 10 g/l lactose, 8.5 g/l yeast extract, 6 g/l corn steep liquor (CSL), 1.2 g/l MgSO_4_.7H_2_O, 1.3 g/l NH_4_Cl, 0.6 g/l CaCl_2_.2H_2_O, pH 5 at 30±2°C and 200 rpm. On the basis of kinetic variables, notably Q_p_ (0.058±0.01^a^ U/g/h), Y_p/s_ (0.308±0.03^ab^ U/g) and q_p_ (0.210±0.032^abc^ U/g fungal biomass/h), *A. oryzae* IIB-6 was found to be a hyper producer of GGH (LSD 0.0345) compared to *A. kawachii* IIB-2. A noticeable enhancement in enzyme activity of over 30% was observed (13.917±1.01 U/ml/min) when the process parameters viz. cultural conditions (pH 5, incubation period 72 h) and nutritional requirements (6 g/l CSL, 9.5 g/l yeast extract, 10 g/l starch, 20 g/l lactose) were further optimized using a 2-factorial Plackett-Burman design. The model terms were found to be highly significant (HS, p≤0.05), indicating the potential utility of the culture (dof~3).

## Background

The enzyme 1,4-α-D-glucan glucohydrolase (GGH, EC 3.2.1.3) is an exo-amylase which cleaves both α-1,4 and α-1,6 glycosidic bonds, yielding β-D-glucose from the non-reducing end of starch polymer chain. GGH degrades starch to glucose in theoretically 100% yields. The reaction rate decreases with the decreasing chain length of the dextrin substrate. The enzyme is also capable of catalysing a reverse of the normal hydrolysis reaction to produce mainly maltose and isomaltose (Rangabhashiyam et al. 2011). It has wide range of applications in industries for the production of dextrose, high-fructose corn syrup (HFCS) and ethanol (Rubinder et al. 2002). With the advent of new frontiers in biotechnology, the spectrum of enzyme applications has widened in many other fields, such as clinical, medicinal and analytical chemistries, and in textile, food, detergent, paper, backing, wine, brewing, distilling and fine chemical industries. Earlier, it was considered that plant and animal materials were the best sources of enzymes. Nowadays, however, microbial enzymes are becoming increasingly important for their technical and economic advantages (Kelly and Fogarty 1976; Kumar and Satyanarayana 2009). A diverse group of microorganisms has been reported to produce the enzyme. However, commercial enzyme has traditionally been produced by employing filamentous fungi, since they secrete large quantities of extracellular enzyme. The production of an active enzyme depends on the selection of a suitable mould for the purpose. Fungal GGH contains both starch binding and catalytic binding domains, the former being responsible for activity on raw starch (Karaoglu and Ulker 2006). The soil is known to be a repository of fungal amylase producers; *Aspergillus, Penicillium, Trichoderma, Fusarium* and *Rhizopus* spp. have been isolated and characterized. However, not even a single comprehensive report has appeared in the available literature dealing with the growth kinetics, mycelial morphology and parametric significance analysis through statistical factorial design. Furthermore, it is also imperative to screen useful fungi for manufacturing of desired product (Oshoma et al. 2010).

The methods of cultivation greatly influence the production and properties of the enzyme. The most common methods of production involve either solid state (SSF) or submerged fermentation (SmF). Traditionally, GGH has been produced by the later processes as enzyme production was about 5 fold higher than with the former (Pandey et al. 2000). The conditions of fermentation such as growth period, temperature, pH, agitation and aeration and medium composition greatly affect the enzyme production under SmF. In SmF, the morphology of filamentous microorganisms varies between two extreme forms, pellets and free filaments, depending on culture conditions and the genotype of the strain. According to previous reports, mycelial morphology is crucial to the process of fermentation, not only in relation to the shape of the hyphae themselves and the aggregation into microscopic clumps (micro-morphology), but also in the pelleted form of growth (macro-morphology). However, reports on the preferred morphology are often contradictory since each one of the two extreme forms - pellets vs. filaments - has their own characteristics concerning cell physiology, growth kinetics, nutrient consumption and broth rheology, which can be regarded either as advantages or as drawbacks (Clementi and Rossi 1986; Papagianni 2004). The present study is concerned with screening of mould cultures, isolated from soil, for the production and optimization of cultural conditions for GGH being carried out aseptically from the selected species and their morphological changes. The 2-factorial Plackett-Burman experimental design was used to further identify the significant batch culture conditions influencing enzyme productivity.

## Results and discussion

In the present studies, sixteen different soil inhabited mould cultures were isolated and screened for GGH production under SmF (Table 1). The enzyme activity and DCM ranged from 1.906-12.675 U/ml/min and 7.236-15.803 g/l, respectively. The maximum enzyme activity (12.675±1 U/ml/min) was obtained with IIB-6 and it was identified as *Aspergillus oryzae*. This enzyme value varied significantly (p≤0.05) than the other species except *A. kawachii* (IIB-2). The DCM was 13.045±1.413 g/l while protein content was recorded to be 73.343±1.522 μg/ml. Therefore, the isolates *A. oryzae* IIB-6 and *A. kawachii* IIB-2, being hyper producers of GGH were selected for kinetic comparison. A diversified mycelial morphology was noticed with other cultures however, *A. oryzae* IIB-6 exhibited mixed kind of mycelia. The product yield was analysed independent of mycelial morphology or pellet size, biomass concentration and protein content as reported by Kelly and Fogarty (1976). Razzaque and Ueda (1978) worked on *Aspergillus* spp. and were also selected one strain of *A. oryzae* RIB-40 as the best GGH producer. Similarly, ten strains of *A. flavus*, two strains of *A. tamarii* and one of each *A. niger* and *A.awamori* were found to be promising producer among all isolates of soil of Kusmi forest (Morya and Yadav 2009). Fourteen *Aspergillus* isolates from soil were chosen as hyper producers by Abdalwahab et al. (2012).

**Table 1:** Screening of different mould cultures for GGH production in submerged fermentation*

Mould cultures		Protein content (μg/ml)	Enzyme activity (U/ml/min)	DCM (g/l)	Mycelial morphology**	Cited bibliography
Isolated strains	Coding					
*Aspergillus* spp.	IIB-1	30±0.997	8.831±0.974	13.131±0.612	Dumpy mass	Papagianni (2004)
*Aspergillus kawachii*	IIB-2	30.341±0.574	12±0.987	12.243±0.926	Large pellets	Shoji et al. (2007)
*Chochliobolus* spp.	IIB-3	64.672±0.521	2.341±0.936	7.232±0.231	Gelatinous	Norouzian et al. (2006)
*Aspergillus niger*	IIB-4	73.675±0.525	4.343±0.986	12.523±0.824	Viscous	Kelly and Fogarty (1976)
*Aspergillus flavus.*	IIB-5	16.671±0.572	11.523±0.525	12.161±0.632	Mixed	(Gomes et al. 2005)
*Aspergillus oryzae*	IIB-6	73.343±0.524	12.673±0.998	13.044±0.413	Mixed	Kassim (1983)
*Arthroderma* spp.	IIB-7	61.661±0.521	1.902±0.523	10.523±0.151	Intermediate pellets	Norouzian et al. (2006)
*Fusarium oxysporum*	IIB-8	29±0.997	2.724±1.082	15.812±0.145	Fine pellets	Karaoglu and Ulker (2006)
*Trichoderma viride*	IIB-9	12±0.996	1.948±1.309	14.343±0.154	Dumpy mass	Karaoglu and Ulker (2006)
*Aspergillus fumigatus*	IIB-10	16.342±0.522	3.839±0.521	12.831±0.115	Fine pellets	Goto et al. (1998)
*Alternaria alternata*	IIB-11	16.673±0.521	5.343±0.997	16.34±0.123	Dumpy mass	Norouzian et al. (2006)
*Rhizopus* spp.	IIB-12	73±0.641	10.185±0.523	13.365±0.151	Viscous	Morita et al. (1998)
*Penicillium italicum*	IIB-13	143±0.998	2.563±0.524	13.845±0.123	Viscous	Nandi and Mukherjee (1989)
*Aspergillus candidus*	IIB-14	12.672±1.052	7.943±0.932	10.232±0.221	Fine pellets	Narayanan and Ambedkar (1993)
*Aspergillus awamori*	IIB-15	10±0.997	4.684±0.975	8.634±0.152	Small pellets	Bertolin et al. (2003)
*Aspergillus foetidus*	IIB-16	10±0.993	5.362±0.521	9.823±0.161	Small pellets	Michelena and Castillo (1984)

* Incubation was carried out for 72 h with agitation intensity 200 rpm at temperature and pH 30±2°C and 5, respectively. ** Foundation of fungal macro-morphology have been laid down in methods section. Y-error bars indicate the standard deviation (±S.D) among the three parallel replicates. The values in each set differ significantly at p≤ 0.05.

The kinetic parameters viz. specific growth rate (μ), product formation parameters (Q_p_, Y_p/s_, Y_p/x_, q_p_) and substrate consumption variables (Y_x/s_, Q_s_, q_s_, Q_x_) were compared for two hyper producing isolates i.e., *A. kawchii* IIB-2 and *A. oryzae* IIB-6 (Table 2). The comparison of Q_s_ (g biomass/L/h) for enzyme productivity demonstrated that isolate IIB-6 has a higher value for volumetric rate of substrate consumption (Q_s_=0.268±0.03^a^ g/l/h) than IIB-2 (0.212±0.021^bc^ g/l/h). A several fold (~10) improvement in terms of volumetric enzyme productivity was noted with the former at all the rates examined. Although IIB-2 achieved a higher value (Y_x/s_=1.124±0.221^ab^ g DCM/g) than IIB-6 (1.065±0.113^bc^ g DCM/g), however the later demonstrated a significant improvement (p≤0.05) in volumetric rate of product formation. In addition, when both of the mould isolates were monitored for specific rate constant, IIB-6 gave higher values for q_p_ (greater than 45% improvement). Hence, *A. oryzae* IIB-6 exhibited an overall 5 to 8 fold improvement in the values for Q_p_, Y_p/x_, Y_p/s_ and q_p_ over the *A. kawchii* IIB-2 (*LSD*~0.034) which is highly significant (HS) and this was supported by the findings reported by (Pirt 1975). Goto et al. (1998) found that nutritional parameters influence the substrate consumption rate, specific growth rate and subsequent GGH productivity. The kinetic values of the enzyme demonstrate that GGH is an exo- rather than an endo- mode as it has higher affinity for the starch rather than other oligo- or polysaccharide polymers.

**Table 2:** Comparison of various kinetic parameters for GGH productivity by*A. kawachii*(IIB-2) and*A. oryzae*(IIB-6) at 72 h of fermentation*

Kinetic parameters	Hyper producing fungal isolates	
	IIB-2	IIB-6
Specific growth rate		
μ (h^-1^)	0.212±0.121^ab^	0.196±0.023^a^
Enzyme production variables		
Q_p_ (U/ml/h)	0.032±0.022^cde^	0.058±0.012^a^
Y_p/s_ (U/ml/g)	0.124±0.0122^def^	0.308±0.031^ab^
Y_p/x_ (U/ml/g)	1.625±0.051^defg^	2.455±0.552^a^
q_p_ (U/g fungal biomass/h)	0.008±0.002^de^	0.210±0.032^abc^
Substrate consumption variables		
Y_x/s_ (g fungal biomass/g)	1.124±0.23^ab^	1.065±0.112^bc^
Q_s_ (g/l/h)	0.212±0.022^bc^	0.268±0.032^a^
q_s_ (g/g fungal biomass/h)	0.125±0.012^bcd^	0.195±0.051^ab^
Q_x_ (g fungal biomass/l/h)	0.142±0.021^bc^	0.216±0.021^a^
Least significant difference (LSD)	0.087	0.034
Significance level <p>	S	HS

* Kinetic parameters: μ (h^-1^)=specific growth rate, Q_p_=U/ml/h, Y_p/s_=U/ml/g substrate consumed, Y_p/x_=U/ml/g fungal biomass formed, q_p_=U/g fungal biomass/h, Y_x/s_=g fungal biomass/g substrate utilized, Q_s_=g substrate consumed/l/h, q_s_=g substrate consumed/g fungal biomass/h, Q_x_=g fungal biomass formed/L/h. HS denotes ‘highly significant’ while *S* stands for ‘significant’ values. LSD represents ‘least significant difference’. <p> is for probability. ± Indicates standard deviation among three parallel replicates. The values designated by different letters in each row differ significantly at p≤0.05.

Among various fermentation media, maximal GGH production (12.609±0.899 U/ml/min, LSD~2.651) was obtained when M3 was used (Table 3). It was due to the fact that enzyme production is strongly influenced by the organic carbon and nitrogen sources as reported by Bertolin et al. (2003). Furthermore, M3 has an adequate amount of C/N sources compared to M1, M2, M4, and M5. Protein content and DCM were 73.287±1.521 μg/ml (LSD~0.267) and 12.967±0.912 g/l (LSD~1.723), respectively. Mixed mycelial pellets were observed in the broth.

**Table 3:** Evaluation of fermentation medium for GGH production by*A. oryzae*IIB-6 in submerged fermentation*

Media (g/l)**	Initial pH	Solvent (1-L)	Protein content (μg/ml)	Enzyme activity (U/ml/min)	DCM (g/l)	Mycelial morphology	Cited bibliography
M1	4.6	0.01 N HCl	27.671±0.521	3.553±0.353	5.541±0.532	Fine pellets	Shoji et al. (2007)
M2	5.5	Deionized H_2_O	63.544±0.998	9.661±0.713	11.612±0.872	Large pellets	Papagianni (2004)
M3	5	Distilled H_2_O	73.284±1.523	12.623±0.854	12.964±0.985	Mixed pellets	Michelena and Castillo (1984); Malik et al. (2011)
M4	7.2	Phosphate buffer	17.671±0.986	1.852±0.734	3.456±0.634	Intermediate pellets	Singh and Soni (2001)
M5	6.4	Sodium acetate buffer	53.334±0.721	7.171±0.532	8.613±0.998	Fine pellets	Morita et al. (1998); Norouzian et al. (2006)
LSD			0.267	2.651	1.723		

* Incubation was carried out for 72 h with agitation intensity 200 rpm at temperature and pH 30±2°C and 5, respectively.

** Media composition is given in the methods section. The constiuents of optimized medium (M3) were further designed in our labs at IIB and have previously been well exploited.

± indicate the standard deviation (S.D) among the three parallel replicates. The values in each set differ significantly at p≤ 0.05.

**Figure 1:**
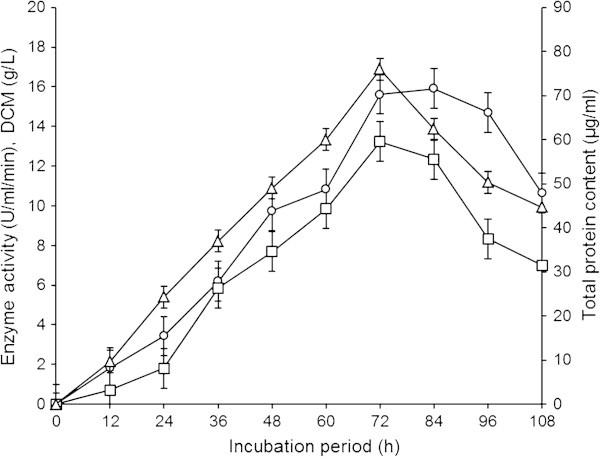
**Time course GGH production by*A. oryzae*IIB-6.** Incubations were carried out at 200 rpm (pH 5) and temperature 30±2°C. Y-error bars indicate standard deviation (±sd) among the three parallel replicates. -□- Enzyme activity (U/ml/min), -○- DCM (g/l), -Δ- Total protein content (μg/ml).

During the time course study (Figure 1), the enzyme production remained very low at 12 h of incubation (0.709±0.989 U/ml/min) and it was demonstrated that the enzyme concentration was correlated with growth of the fungus (Hyland and Clayton 1992). It was increased by increasing the incubation period but upto a certain extent. Maximum enzyme production (13.227±1.521 U/ml/min, LSD~2.268) was achieved 72 h after the inoculation. It was approximately 18.66 fold higher than the enzyme activity obtained at 12 h. The protein content and DCM were 76±1.042 μg/ml (LSD~2.184) and 15.623±1.154 g/l (LSD~0.093), respectively. The mycelial morphology was found as small pellets. Similarly, Morita et al. (1998) obtained maximum enzyme production in 72 h of fermentation. The enzyme activity was gradually decreased when incubation period was prolonged beyond the optimal, it was due to the depletion of nutrients and accumulation of other by products such as proteases which acted on starch binding domains causing the enzyme to lose its activity in the fermented broth. Abdalwahab et al. (2012) reported that *A. awamori*, *A. niger* and *A. tamarii* have shown maximum enzyme production after 48 h while *A. terreus* showed maximum performance of enzyme production after 72 h. In another study, incubation period of 96–120 h was optimized by Kumar and Satyanarayana (2007).

**Figure 2:**
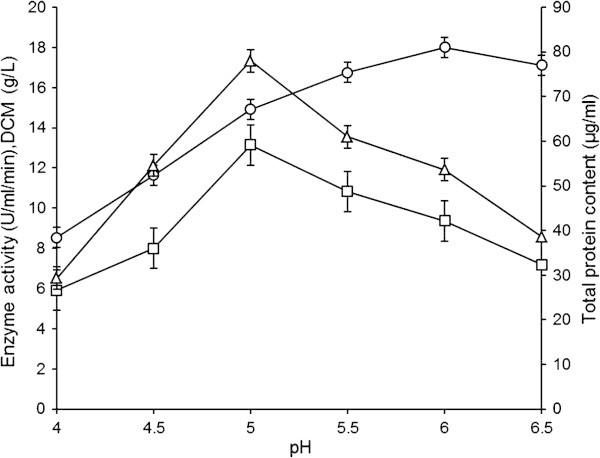
**Effect of initial pH on GGH production by*A. oryzae*IIB-6.** Incubations were carried out for 72 h at 30±2°C and 200 rpm. Y-error bars indicate standard deviation (±sd) among the three parallel replicates. -□- Enzyme activity (U/ml/min), -○- DCM (g/l), -Δ- Total protein content (μg/ml).

GGH activity was not encouraging at pH 4 (5.911±1.031 U/ml/min) be due to the fact that an acidic pH was toxic to the mycelia of *A. oryzae* resulting in the inhibition of GGH production. The maximum enzyme production (13.152±1.021 U/ml/min) was achieved when initial pH was adjusted to 5 (LSD~1.715) and varied significantly than other values (p≤0.05) as depicted in Figure 2. Mixed mycelia were found in the flask. The protein content and DCM were 78±1.723 μg/ml (LSD~2.889) and 14.922±1.061 g/l (LSD~0.507), respectively. Similarly, previous workers also optimized pH 5 for enzyme production (Mishra and Das 2002). Abdalwahab et al. (2012) was found optimum pH for maximum enzyme production to be 6.0 for all isolates (*A. awamori , A. niger* and *A. tamarii*) except for *A. terreus* which gave maximum production at pH 4.0. A sharp decline in enzyme activity (7.183±1.201U/ml/min) was observed at pH 6.5. It was due to the fact that the mould required slightly acidic pH for its growth and subsequent enzyme production as reported earlier by Bertolin et al. (2003).

**Figure 3:**
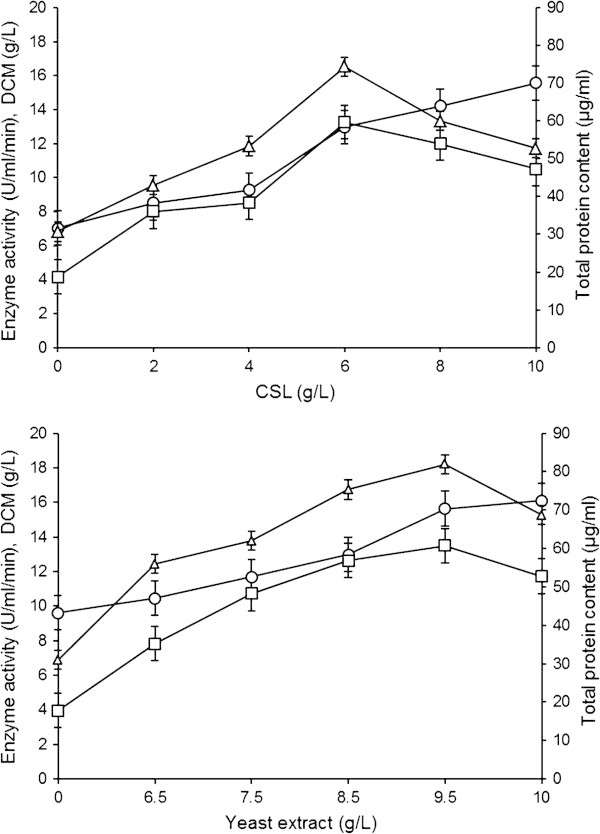
**Effect of different concentrations of CSL and yeast extract as nitrogen sources on GGH production by*A. oryzae*IIB-6.** Incubation were carried out for 72 h (200 rpm) at 30±2°C and pH 5. Y-error bars indicate standard deviation (±sd) among the three parallel replicates. -□- Enzyme activity (U/ml/min), -○- DCM (g/l), -Δ- Total protein content (μg/ml).

The effect of different concentrations of CSL and yeast extract on GGH production are shown in Figure 3. Maximum production was observed to be 13.263±1.501 U/ml/min when 6 g/l CSL was used in the fermentation medium (LSD~7.006), which was nearly 3.02 fold higher than control. Small pellets were observed in the fermented broth. The protein content and DCM were 74.342±1.521 μg/ml (LSD~5.656) and 12.974±1.051 g/l (LSD~0.126), respectively. Kassim (1983) used 30 g/l CSL in the fermentation medium. Therefore, our finding is economically more significant. In contrast to our study, Michelena and Castillo (1984) optimized only 0.8 g/l CSL for better enzyme production. Cherry et al. (2004) who reported that *A. fumigatus* produces high amylase activity with yeast extract. However, Abdalwahab et al. (2012) found that yeast extract affect negatively enzyme production by *A. tamarii* and *A. awamori* while with *A. niger* and *A. terreus* it stimulate the production. In present study, the maximum production (13.489±1.601 U/ml/min, LSD~4.091) was obtained when 9.5 g/l was added into the fermentation medium by *A. oryzae*. The mycelial morphology was observed as large pellets. Protein content and DCM were found to be 81.941±1.501 μg/ml (LSD~1.778) and 15.643±1.115 g/l (LSD~0.175), respectively. Similarly, Kumar et al. (2007) observed maximum enzyme production when 10 g/l yeast extract was used in fermentation medium. Sporangiospores secreted 41% higher GGH titres in shake flasks when 20 g/l yeast extract was used (Oshoma et al. 2010). However, Nandi and Mukherjee (1989) reported that yeast extract (5 g/l) stimulates the production of enzyme.

**Figure 4:**
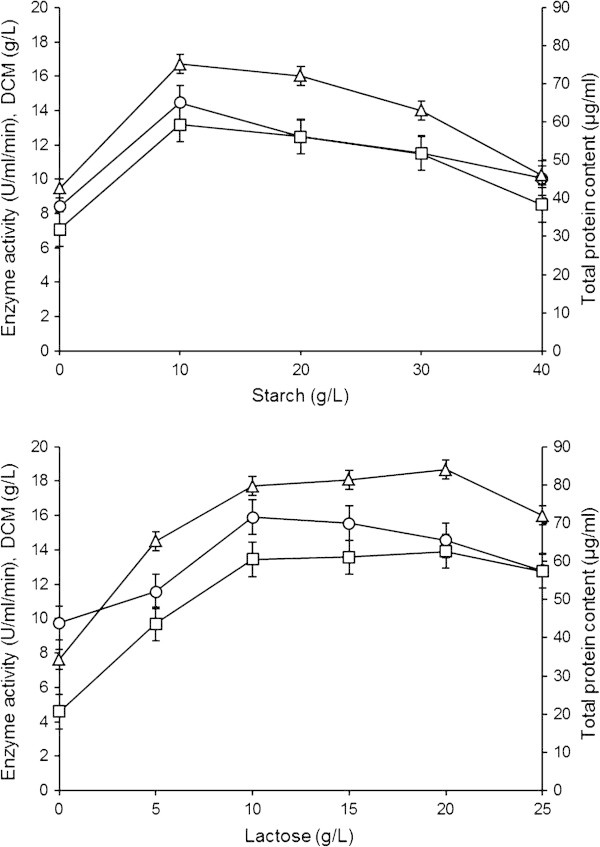
**Effect of different concentrations of starch and lactose as carbon sources on GGH production by*A. oryzae*IIB-6.** Incubation were carried out for 72 h (200 rpm) at 30±2°C and pH 5. Y-error bars indicate standard deviation (±sd) among the three parallel replicates. -□- Enzyme activity (U/ml/min), -○- DCM (g/l), -Δ- Total protein content (μg/ml).

Different concentrations of starch and lactose were added into the fermentation medium to induce better GGH production (Figure 4). The maximum enzyme activity (13.184±0.991 U/ml/min) was noted when 10 g/l starch was used (LSD~1.735) which was about 1.86 fold higher compared to the control. The protein content and DCM were 75.162±1.081 μg/ml (LSD~2.266) and 14.471±1.213 g/l (LSD~0.287), respectively. Mycelial morphology was in the form of small pellets. Similarly, Kunamneni et al. (2005) and Arnthong et al. (2010) reported that enzyme production reached maximal when 10 g/l starch was added in the fermentation medium. As the amount of starch was increased enzyme production was decreased which was due to carbon catabolite repression (Morita et al. 1998). However, other workes reported that 20 g/l rice flour (as a source of starch) concentration seems to be the concentration that gave maximum production of GGH. Above 20 g/l, there was little increase in enzyme production. These results were mostly obvious in the case of *A. niger* and *A. awamori* (Abdalwahab et al. 2012). Previously, Cherry et al. (2004) optimized 40 g/l starch concentration for enzyme production from fungal isolate *A. fumigatus.*

The lactose concentrations (20 g/l) gave maximum enzyme production (13.917±1.012 U/ml/min, LSD~0.636). The mycelial morphology was observed as round pellets of intermediate sized. The protein content and DCM were 84.00±4.05 μg/ml (LSD~2.712) and 14.56±1.24 g/l (LSD~0.507), respectively. Other lactose concentrations (10 and 15 g/l) also induced relatively better enzyme production i.e., 13.461±1.511 U/ml/min and 13.573±0.502 U/ml/min, respectively. However in contrast to our study, Singh and Soni (2001) used 10 g/l lactose to stimulate enzyme production. Similar observations have also been made by Negi and Banerjee (2010) for amylase production by *A. awamori.* Some other workers (Vidya et al., 2012) used lactose at 30 g/l concentration and found to be the best source for maximum amylase production.

The 2-factorial experimental system i.e., Plackett-Burman design was applied to determine the significant process parameters involved in GGH production by the newly isolated mould culture of *A. oryzae* IIB-6 (Table 4). The validation of the model was investigated under the conditions predicted against the responses obtained for enzyme production. A differential correlation was noted between the observed and predicted values as reported by Burkert et al. (2006). The optimal levels of the parameters for improved enzyme production under submerged fermentation (SmF) were incubation period (72 h), initial pH (5), CSL 6, yeast extract 9.5, starch 10 and lactose 20 (g/l). The statistical analyses of the responses for enzyme production were also performed (Table 5). The correlation (1.618*E*+0025) of A, B, C, D and E for F values depicted that the model was highly significant (HS, p≤0.05). Correspondingly, the lower probability values indicated that the model terms are statistically valid. The analysis of linear, quadratic and interaction coefficients were performed on the batch culture results which highlighted that enzyme production was a function of the independent parameters (Ahuja et al. 2004) Lactose used as a sole carbon source (degree of freedom 3) has an important physiological role in the improvement of enzyme activity (Burkert et al. 2006). According to these results, the fungal strain of *A. oryzae* IIB-6 could be considered as an organism of choice for GGH productivity.

**Table 4:** Application of Plackett-Burman design at various process parameters (designated by different captions) for GGH production by*A. oryzae*IIB-6*

Process parameters identified through 2-factorial design						Enzyme activity (U/ml/min)	
Cultural conditions		Nitrogen sources		Carbon sources			
Incubation period (h)^A^	Initial pH^B^	CSL conc. (g/l)^C^	Yeast extract (g/l)^D^	Starch conc. (g/l)^E^	Lactose conc. (g/l)^F^	Observed	Predicted
24	4	*sna*	6.5	*sna*	5	6.252	8.245
36	4.5	2	7.5	*sna*	10	8.654	9.558
48	4.5	4	8.5	10	15	10.435	12.126
72	5	6	9.5	10	20	13.917	13.756
72	5.5	6	10	20	20	12.544	13.642
84	6	8	10	30	25	11.006	10.865

*The different letters represent significant process parameters for GGH fermentation. Statistical analysis of the model was based on 2-factorial experimental design. The abbreviation ‘*sna*’ means that specific nutritional source (C/N) was not added into the fermentation medium (M3).

**Table 5:** Statistical analysis of 2-factorial experimental design at various significant process parameters for GGH production by*A. oryzae*IIB-6*

Significant process parameters	Sum mean values	F-value	Degree of freedom	Probability <p>
A	4.133	7.628	1	0.078
B	7.415	10.784	1	0.065
C	8.768	12.105	1	0.057
D	10.225	14.482	2	0.052
E	12.688	15.295	3	0.034
F	12.112	14.044	2	0.025
Correlation	1.618*E*+0025			

*CM – 19.24; *R*^*2*^ – 0.238. The cap-letters represent significant process parameters (incubation period, initial pH, CSL conc., yeast extract conc., starch conc., lactose conc.) for enzyme production.

## Conclusions

A soil-inhabited mould isolate *A. oryzae* IIB-6 was identified as a hyper producer of 1,4-α-D-glucan glucohydrolase (GGH) in submerged fermentation (SmF). M3 as a basal medium gave better GGH yield at 30±2°C (200 rpm). The cultural conditions such as pH 5 and incubation period (72 h) were also optimized. Among the carbon and nitrogen sources, lactose (20 g/l) and yeast extract (9.5 g/l) raised the enzyme activity to a maximal of 13.91 U/ml/min. The values of kinetic variables, notably Q_p_ (0.058±0.011^a^ U/ml/h), Y_p/s_ (2.455±0.551^a^ U/ml/g) and q_p_ (0.210±0.032^abc^ U/g fungal biomass/h) demonstrated that the isolated mould culture has a faster growth rate and subsequently a higher enzyme production capability (*LSD*~0.034). An overall improvement of more than 30% in terms of enzyme activity was accomplished when the significant process parameters were determined after Plackett-Burman design. The value of correlation (1.618*E*+0025 with dof~3) depicted that the model terms are highly significant (HS, p≤0.05). However, enzyme characterization is in progress prior to scale up studies.

## Methods

The chemicals and reagents used in this study were of analytical grade and procured directly from Sigma (USA), BDH (UK) and Fluka (Switzerland).

### Isolation and preliminary screening of mould cultures

The soil samples were collected from various localities of Doha (Qatar) in sterilized polythene bags. Each sample was diluted by serial dilution method. One millilitre of appropriately diluted soil suspension (10^− 5^, 10^− 6^) was plated on starch agar medium (10 g/l raw corn starch, 1.496 g/l KH_2_PO_4_, 1 g/l MgSO_4_.7H_2_O, 1 g/l NaNO_3_, 20 g/l agar, pH 4.8 and sterilized at 121°C, 15 lbs/in^2^ pressure for 15 min) using pour plate method. The plates were incubated at 30±2°C for 72 h and subsequently flooded with iodine solution (2 g/l iodine, 4 g/l potassium iodide prepared in deionized water). The zone of clearance around the microbial growth indicated GGH activity. The initial colonies of mould cultures showing bigger zones (~2 mm^2^) of starch hydrolysis in the plates were picked up and transferred to potato dextrose agar (PDA) slopes (pH 5.6) aseptically and then incubated at 30±2°C for 4–6 days until optimal growth. The slant cultures were stored at 4°C in a mini cold lab (430D, Gallenkamp, London, UK) and renewed at least twice a month.

### Identification of mould isolates

The fungal isolates were identified morphologically using a scotch tape of approximately 1cm in length. The sticky end was placed over the fungal culture to pick up mycelia and other reproductive structures of fungi as reported by Harris (2000). It was placed upwards on a microscope slide. A drop of 0.5 g/l trypan blue (prepared in lactophenol) was added. A coverslip was placed over the slide culture and then visualized at 40X under a compound microscope. The identified mould cultures were confirmed after (Onion et al. 1986).

### Inoculum preparation

A volume of 10 ml of sterilized 0.5 g/l di-acetyl ester of sodium sulpho succinic acid (monoxal OT) were aseptically transferred to a slant culture having optimal conidial growth. The clumps of spores were broken with the help of a sterile inoculating wire loop. A homogeneous suspension was made by gently shaking the tube. The spore count was made by a haemocytometer (130M, Neubyeur, Munich, Germany) and found to be 1.2×10^7^ CFU/ml.

### Fermentation procedure and critical phases

Shake flask fermentation technique was employed for 1,4-α-D-glucan glucohydrolase (GGH) production under submerged fermentation (SmF) technique. One milliliter spore suspension was transferred to the individual 250 ml Erlenmeyer flasks containing 50 ml sterilized (at 121°C, 15-lbs/in^2^ pressure for 15 min) M3 liquid medium (found optimal). The initial pH was adjusted to 5. All the microbial fermentations were carried out in a rotary shaking incubator at 30±2°C, 200 rpm for 72 h. The experiments were run parallel in a set of three replicates.

### Fermentation media

Following media were evaluated for GGH production during the course of study,

M1. 30 g/l wheat bran 30, 1-L 0.01 N HCl, pH 4.6.

M2. 10 g/l starch, 5 g/l lactose, 10 g/l nutrient broth, 2 g/l (NH_4_)_2_SO_4_, 2 g/l CaCl_2_.2H_2_O, 1-L deionized water, pH 5.5.

M3. 20 g/l starch, 10 g/l lactose, 8.5 g/l yeast extract, 6 g/l corn steep liquor, 1.2 g/l MgSO_4_.7H_2_O, 1.3 g/l NH_4_Cl, 0.6 g/l CaCl_2_.2H_2_O, 1-L distilled water, pH 5.

M4. 3 g/l yeast extract, 20 g/l peptone, 0.05 g/l MgSO_4_.7H_2_O, 0.2 g/l CaCl_2_.2H_2_O, 0.1 g/l FeSO_4_, 1-L phosphate buffer, pH 7.2.

M5. 10 g/l starch, 10 g/l nutrient broth, 2.4 g/l (NH_4_)_2_SO_4_, 5 g/l CaCl_2_.2H_2_O, 1-L sodium acetate buffer, pH 6.4.

### GGH isolation from fermented mash culture

The fermented broth was filtered through an oven dried (at 102°C for 15 min) pre-weighed Whatman filter paper No. 1. The mycelial morphology was observed. The clear filtrate was used for enzyme and protein assay, while cell mass was used to calculate dry weight.

### Determination of mycelial morphology

Mycelial morphology was observed at macro level (Onion et al. 1986). Mycelia were categorized on the basis of their form and size as follow: Fine pellets (round clumps with diameters between 1–1.5 mm); small pellets (round clumps with diameters between 3–3.5 mm); intermediate pellets (round with diameters between 4–4.5 mm); large pellets (round clumps with diameters between 6–6.5 mm); mixed (a mixture of all previous four forms); viscous (a thick mixture of small and fine pellets with some free filaments); gelatinous (gel like mixture of fine pellets and filaments) and dumpy mass (irregular single mass with variable mycelial sizes).

### Analytical techniques

The cell mass left in the pre-weighed filter paper was washed twice with distilled water and oven dried at 102°C for 2 h. The dry cell mass (DCM) was calculated by subtracting the weight of filter paper from the final weight and converted to g/l. GGH was assayed according to the method of Caldwell et al. (1968). One millilitre of enzyme (diluted to 10^−3^ times) and 1 ml of substrate (50 g/l Litner’s soluble starch solution in 0.05 M sodium acetate buffer, pH 5) was incubated at 60°C for 60 min with a constant stirring speed of 100 rpm. The amount of reducing sugar liberated was determined using 3, 5-dinitrosalicylic acid (DNS) reagent by measuring *A*_546nm_ on a spectrophotometer against glucose as the standard. “One unit of GGH activity was the amount of enzyme that liberates 1 mg of reducing sugar (as glucose) under the specified assay conditions”. The sugar released was then converted into U/ml/min. Protein concentration was estimated in the filtrate by the method of Bradford (1976) with crystalline bovine serum albumin as the standard. The protein content was monitored by measuring *A*_595nm_.

### Secondary screening of isolated mould cultures by SmF technique

Sixteen different mould cultures (coded as IIB-1 to IIB-16) of genera *Aspergillus*, *Alternaria*, *Arthroderma, Fusarium, Trichoderma, Penicillium, Rhizopus* and *Chochlobolus* spp. were screened for GGH production (Table 1). All the cultures were tested in triplicates by incubating the fermentation medium (M3 optimal) at pH 5, 30±2°C, 200 rpm for 72 h.

### Parametric analysis by kinetic study

Kinetic variables were studied according to the procedure of (Pirt 1975). The values for specific growth rate i.e., μ (h^-1^) were calculated from the plots of ln(X) versus time of fermentation. The growth yield coefficient (Y_x/s_) was calculated as the dry cell mass divided by the amount of saccharide utilized during the course of fermentation. The product yield coefficients namely Y_p/s_ and Y_p/x_ were determined by using the relationships Y_p/s_=dP/dS and Y_p/x_=dP/dX, respectively. The volumetric rates for substrate utilization (Q_s_) and product formation (Q_p_) were determined from the maximum slopes in plots of substrate utilized and GGH produced versus the time of fermentation (dt). The volumetric rate for biomass formation (Q_x_) was calculated from the maximum slope in a plot of cell mass formation versus incubation time period. The specific rate constants for product formation (q_p_) and substrate utilization (q_s_) were determined by the equations q_p_=μ×Y_p/x_ and qs=μ×Y_s/x_, respectively. Further, the specific rate for cell mass formation (q_x_) was, calculated by multiplying the specific growth rate (μ) with the growth yield coefficient (Y_x/s_).

### Determination of significant batch culture conditions

Different fermentation media (M1, M2, M3, M4, M5) were evaluated for GGH production by *A. oryzae* IIB-6 (Table 3). All media were incubated at 30±2°C, 200 rpm for 72 h. The time course profile for GGH production in shake flasks was studied by incubating M3 medium. Incubation was carried out for 12–108 h at 30±2°C (200 rpm). The optimal initial pH for enzyme production was measured by incubating the fermentation medium for 72 h under a narrow pH range (4–6.5). The optimum concentrations of nitrogen and carbon sources were also investigated. The effects of different concentrations of CSL (2–10 g/l) and yeast extract (6.5-10 g/l) as nitrogen sources on GGH production were measured and compared with the control (run parallel). Different concentrations of starch (10–40 g/l) and lactose (5–25 g/l) as carbon sources were employed to study their effects on enzyme production. The experiments of C/N sources were conducted separately in triplicates at pH 5 for 72 h.

### Statistical analysis and application of Plackett-Burman experimental design

Duncan’s multiple range tests (Spss-16, version 9.5) were applied under one-way analysis of variance (I-ANOVA) and the treatment effects were compared after (Snedecor and Cochran 1980). Significance was presented in the form of probability (<*p>*) values. The significant batch culture conditions affecting improved GGH productivity were identified using a 2-factorial system i.e., Plackett-Burman experimental design (Ahuja et al. 2004) The variables were denoted at two widely spaced intervals and the effect of individual parameters on enzyme production was calculated by the following equations,12

In Eq. I, E_ο_ is the effect of first parameter under study while M+ and M− are responses of enzyme by the selected fungal isolate. N is the total number of optimizations. In Eq. II, E is the significant parameter, β_1_ is the linear coefficient, β_2_ the quadratic coefficient while β_3_ is the interaction coefficient among significant process parameters.

## Abbreviations

GGH: 1,4-α-D-glucan glucohydrolase
IIB: Institute of Industrial Biotechnology
CSL: Corn steep liquor
HS: Highly significant, dof, degree of freedom
SmF: Submerged fermentation
PDA: Potato dextrose agar
DCM: Dry cell mass
DNS: Dinitrosalicylic acid
ANOVA: Analysis of variance.
